# Usnic Acid-Loaded Polymeric Micelles: An Optimal Migrastatic-Acting Formulation in Human SH-SY5Y Neuroblastoma Cells

**DOI:** 10.3390/ph15101207

**Published:** 2022-09-29

**Authors:** Marzia Vasarri, Linda Ponti, Donatella Degl’Innocenti, Maria Camilla Bergonzi

**Affiliations:** 1 Department of Experimental and Clinical Biomedical Sciences “Mario Serio”, Viale Morgagni 50, 50134 Florence, Italy; 2 Department of Chemistry, University of Florence, Via Ugo Schiff 6, Sesto Fiorentino, 50019 Florence, Italy

**Keywords:** usnic acid, polymeric micelles, solubility, SH-SY5Y, wound-healing assay, cell migration, metalloproteinases

## Abstract

Usnic acid (UA) is one of the most abundant and common metabolites of lichens, known for its numerous pharmacological properties. Nevertheless, it presents some criticalities that severely limit its use in therapy: poor solubility in water and significant hepatotoxicity. Soluplus and Solutol HS15 and D-α-Tocopherol polyethylene glycol 1000 succinate (TPGS) were employed to develop polymeric micelles (UA–PM). The chemical and physical properties of the system were characterized, including the size, homogeneity, zeta potential, critical micellar concentration (CMC), encapsulation efficiency (EE%), and in vitro release. The freeze-drying process was considered to prevent agglomeration and improve the stability of the formulation. The stability of the micelles and the freeze-dried product (UA–PML) was also evaluated. The anti-migratory activity of UA and UA–PM was evaluated in human neuroblastoma SH-SY5Y cells using the wound healing assay. Their effect on the activity of metalloproteinases (MMP)-2/9 involved in the migration process of cells was verified by gelatin zymography. The optimized UA–PM contained Soluplus, Solutol HS15, and TPGS in a 1:4:0.5 weight ratio and increased the aqueous solubility to about 150-fold solubilized, solubilizing 0.5 mg/mL of UA. UA–PM has a small size (45.39 ± 0.31 nm), a polydispersity index (PDI) of 0.26 ± 0.01, and an EE% of 82.13 ± 5.57%. The colloidal dispersion was stable only for 9 days at 4 °C, while the freeze-drying process improved the stability for up to 30 days. UA was released for a prolonged period during the in vitro release study. The in vitro cell-based experiments showed that UA–PM (0.2 µg/mL) inhibited SH-SY5Y cell migration and the gelatinolytic activity of MMP-2/9 in culture media, while free UA at the same concentration exerted no biological activity. This study demonstrates that polymeric micelles are an excellent formulation for UA to manifest inhibitory action on neuroblastoma cell migration.

## 1. Introduction

In addition to plants, lichens are a rich source of bioactive compounds and have been used since ancient times in folk medicine to treat various diseases worldwide [1]. Lichens and their secondary metabolites possess important anti-tumorigenic properties [2] due to their antioxidant, antiproliferative, cytotoxic, pro-apoptotic, anti-invasive, and anti-migratory abilities [3].

**Figure 1:**
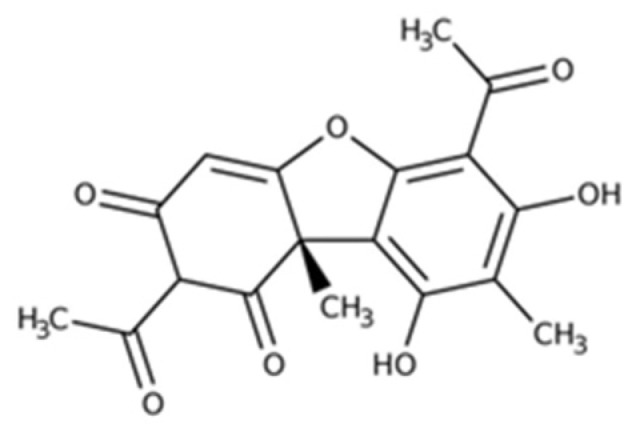
Chemical structure of usnic acid.

Usnic acid (UA; 2,6-diacetyl-7,9-dihydroxy-8,9b-dimethyl-1,3(2H,9bH)-dibenzofurandione) is an abundant metabolite commonly found in a variety of lichen species such as *Usnea*, *Cladonia*, *Lecanora*, and *Alectoria* (Figure 1).

This natural compound might be of great relevance for pharmacological and clinical applications because of its different biological and physiological properties. It is known for its antibacterial and antiparasitic potentials, antiviral, antioxidant, anti-inflammatory, analgesic, and UV protection activities. This molecule is of potential interest for cancer therapy due to its antimitotic and antiproliferative action [4,5,6,7,8].

Neuroblastoma (NB) is a fairly common extracranial solid tumor occurring at pediatric age, with a clinical course dependent on the tumor biology [9,10].

NB is classified as low-risk if the tumor regresses spontaneously or with conventional treatment options and as aggressive high-risk NB if the disease is refractory or recurrent with widespread metastases at the time of diagnosis [11]. In cancer patients, metastasis is the leading cause of death and depends on the invasive ability of the cells that break away from the primary tumor and migrate to other regions to form a secondary tumor [12,13]. In high-risk NB, cancer cells acquire the ability to penetrate the basement membrane of blood vessels by destroying the extracellular matrix (ECM) components to metastasize. In this process, MMP-2 and MMP-9, also known as gelatinases, have a key role in invasion and metastasis formation [14]. Therefore, to date, intervening in cancer patients by blocking cancer cell migration and thus preventing metastasis formation remains a challenge in cancer therapy [15].

In an effort to address the disadvantages of conventional cancer therapies, natural compounds are still widely studied for their anticancer properties in order to develop new complementary therapies [16].

The literature proposes UA as a good candidate for anticancer therapy due to its inhibitory activity of cancer cell migration [17,18,19]. However, UA is an extremely hydrophobic molecule with a water solubility of 3 μg/mL [20,21], which limits its application as a therapeutic agent. This compound is an active ingredient for the disinfection of skin, burns, wounds, or fungal skin infections in topical application products currently on the market.

Nano- and micro-sized colloidal carriers have been proposed as drug delivery systems to increase solubility and ameliorate the therapeutic index, improving the efficacy and reducing the hepatotoxicity of UA after systemic administration [22].

Liposomes [23,24,25], nanoemulsions [26], polymeric micro- and nano-carriers [27,28,29,30], nanofibers [31], and non-organic nanocarriers [32,33,34] were applied to improve UA’s pharmacological profile as an antimicrobial, antitumor, wound-healing, antioxidant, and anti-inflammatory drug.

In this study, for the first time, polymeric micelles were proposed as a nanocarrier to increase UA aqueous solubility (UA–PM). Polymeric micelles (PM) offer numerous clinical applications, such as the protection of the encapsulated drug and the solubilization of poorly soluble drugs. Here, the polymers selected for the PM preparations were Solutol HS15, Soluplus, and D-α-Tocopherol polyethylene glycol 1000 succinate (TPGS), three tensides with high solubilizing properties [35,36,37,38,39,40] that can promote permeation across biological membranes, such as the intestinal, nasal, buccal mucosae, skin, and corneum barriers, they also inhibit P-gp [35,41,42,43]. In a previous study, the authors encapsulated thymoquinone in the Soluplus–Solutol micelles, improving their solubility and anti-migratory activity [44]. In this work, empty and UA–PM were physically and chemically characterized, and the UA in vitro release from the carrier was also considered. To ameliorate the storage stability, the authors tested the stability of micelles as colloidal dispersion for 30 days, and they considered the freeze-drying process as a method to transform the dispersion into a solid product (UA–PML) with better stability over time. Freeze-drying was used to remove the water and increase or protect the stability of the micelles in favor of less transportation costs.

The effect of UA and UA–PM on cell migration and MMP-2/9 gelatinolytic activity were evaluated in SH-SY5Y human neuroblastoma cells, thus verifying the potential enhancement of the biological activity of UA delivered into the PM.

## 2. Results and Discussion

### 2.1. Physical and Chemical Characterization of PM and UA–PM

PM has a core–shell structure due to the self-assembly of the amphiphilic block copolymers when added to an aqueous solvent. They have a hydrophobic core, capable of encapsulating poorly water-soluble compounds, and an external hydrophilic portion that prolongs the retention time in the bloodstream, preventing opsonization and absorption by macrophage phagocytosis and can be functionalized for drug targeting [42,45,46,47]. In this study, Soluplus, Solutol, and TPGS, which are used as solubilizers and absorption enhancers in the food and pharmaceutical industries, were selected as PM constituents to ameliorate the solubility and permeability of UA. The PM and UA–PM were prepared according to the hydration method of the lipid film using a Soluplus:Solutol:TPGS in a 1:4:0.5 weight ratio. The lipid film of the PM appeared transparent and limpid, while that of the UA–PM was slightly yellow due to the color of the active ingredient. The water solubility of UA was about 3 μg/mL, and it increased more than 150 times to 0.5 mg/mL with the formulation in PM. The physicochemical characterization of the empty and loaded micelles is reported in Table 1.

The interaction of UA and PM was also investigated by UV-Vis absorption. The UV analyses were recorded for the UA solution, UA–PM, and PM alone (Supplementary Materials, Figure S1). The blank micelles did not express evident absorbance, whereas the UA solution exhibited significant absorbance at 280 nm. After UA was encapsulated in micelles, the absorption peak of UA decreased, evidence of the encapsulation of UA into micelles [48,49].

PM and UA–PM had similar sizes of about 50 nm. The encapsulation of UA maintained a homogeneous system, as evidenced by the PDI value. The EE% was 80.04%, indicating the high affinity of the molecule for the hydrophobic core of the micelles.

**Figure 2:**
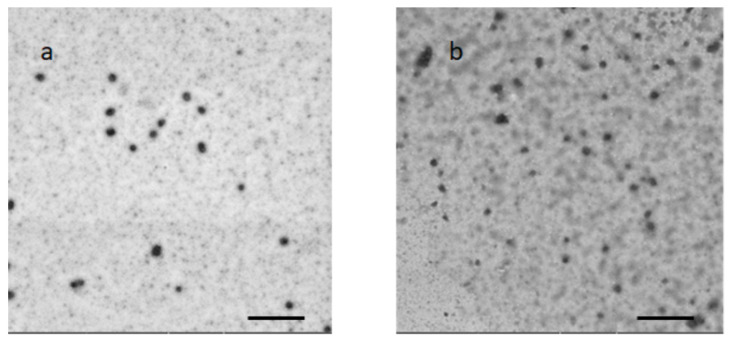
UA–PM (**a**) and UA–PML (**b**) TEM analysis Bar: 500 nm.

TEM analysis of UA–PM confirmed the DLS results, highlighting structures around 50 nm. (Figure 2a).

The preparation of PM for the UA delivery has never been reported before in the literature, and the physical and chemical parameters of the system showed a very promising formulation to improve the biopharmaceutical characteristics of this compound. The theoretical critical micellar concentration (CMC) value for PM is 125 mg/L [50].

### 2.2. In Vitro Drug Release Study

**Figure 3:**
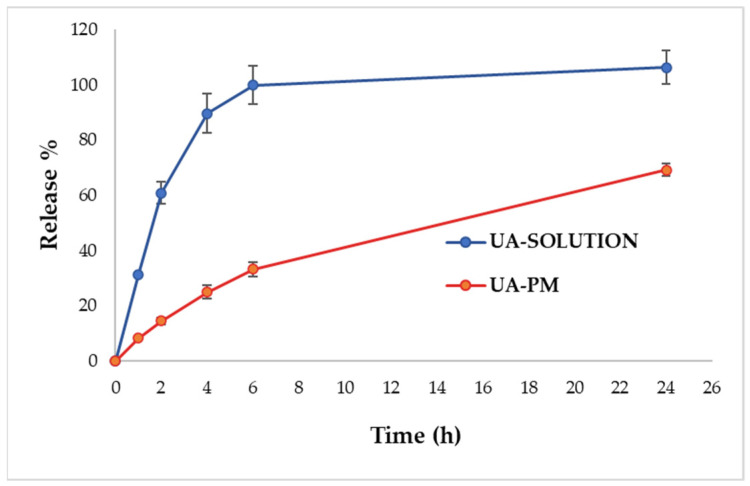
In vitro release profiles of UA from solution and from UA–PM in PBS:EtOH 70:30. Data are reported as mean ± SD of *n* = 3 experiments.

The release profiles of UA from the micelles and from the solution in PBS:EtOH 70:30 at 37 °C are shown in Figure 3. The profile of UA–PM demonstrated a slow release compared with the solution, providing a prolonged release. The results indicated that the release of UA from the solution through the dialysis membrane was much faster, with a fast release during the first 2 h and approximately 100% of the drug released within 6 h. On the contrary, the amount of UA released from the PM in 6 h was around 30%. After 24 h, the release reached 70% without a burst release. The slow release of the drug obtained with PM might be due to the high hydrophobic character of the PM core, which delayed the diffusion of water into the core, preventing the subsequent diffusion of UA [47,51,52,53].

### 2.3. Storage Stability Study

**Figure 4:**
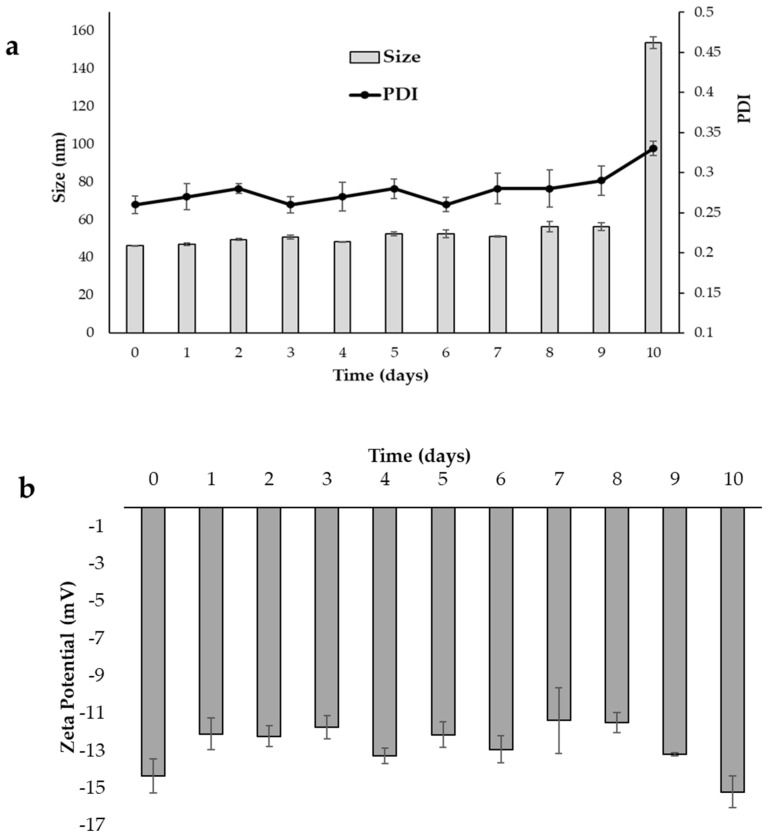
UA–PM as colloidal dispersion after 10 days of storage at 4 °C. (**a**) Particle size and polydispersity index (PDI). (**b**) Zeta potential. Data are shown as mean ± SD (*n* = 3).

**Figure 5:**
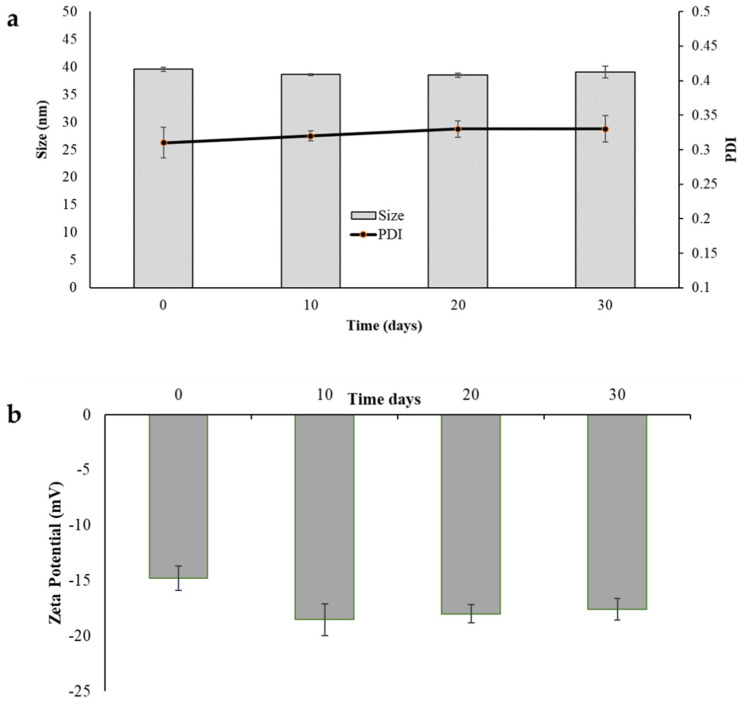
Freeze-dried micelles (UA–PML) stored for 30 days at 25 °C. (**a**) Particle size and polydispersity index after reconstitution of the colloidal dispersion (PDI). (**b**) Zeta potential after reconstitution of the colloidal dispersion. Data are shown as mean ± SD (*n* = 3).

The development of a formulation also implies an adequate stability study that includes checking the parameters susceptible to change during storage under different conditions, which could affect the quality of the product. The stability study of PM consisted of visual control and analytical evaluation of UA content and physical parameters such as sizes, PDI, and zeta potential. For this purpose, the micellar dispersion was stored at 4 °C for 30 days, while the freeze-dried product (UA–PML) at room temperature for 30 days. In the 1-month stability test, no sedimentation or phase separation was observed in the case of dispersion. The freeze-dried powder was stable without any collapse of the solid. The EE, particle size, PDI, and zeta potential of UA–PM and the freeze-dried micelles (UA–PML) were reported, and they are shown in Figure 4 and Figure 5. The UA–PM as colloidal dispersion appeared physically stable for 9 days, with sizes ranging from 46.20 to 56.38 nm, PDI values between 0.26 and 0.29 (Figure 4a), and a zeta potential between −14.34 and −15.19 mV (Figure 4b). However, on day 10, a noticeable increase in size (153.91 nm) is evident, indicating an aggregation phenomenon. The EE% of UA in the micelles decreases by 21% after 3 days and becomes 47% after 10 days, indicating a destabilization of the system with drug leakage from the carrier, already evident from the physical stability results.

To avoid aggregation and/or chemical instability, the authors considered the freeze-drying process as a method to transform the colloidal dispersion into a solid with better stability over time. Freeze-drying is used to remove water and increase or protect the stability of the micelles in favour of fewer transportation costs. Then, at predetermined time intervals, the solid was redispersed in water, and the reconstituted micelles were physically and chemically characterized. The physicochemical parameters of the UA–PML were comparable with those of dispersion, as evidenced by data at the beginning of the test (sizes 40 nm vs. 46 nm; PDI 0.31 vs. 0.26; zeta potential −14.79 vs. −14.24) (Figure 5a,b). The unchanged dimensions were also confirmed by the TEM image (Figure 2b). After 30 days of storage, the system maintained unchanged physical characteristics, and only a 10% reduction in EE% was obtained. Upon storage and transportation, drug leakage may take place due to the diffusion of the drug outward from the micelles due to temperature fluctuations. One approach to overcome such a problem is the lyophilization of the micellar solution to remove the water completely. Overall, the results suggested that lyophilization is a good method to preserve the micellar formulation, improving its chemical and physical stability.

Furthermore, by means of a visual check, the freeze-dried product showed easy reconstitution without the presence of any sediment. The re-dispersibility of UA–PML was also evaluated according to the previously reported method [54]. The results are expressed as the particle size ratio of loaded micelles after reconstitution (S2) and before lyophilization (S1). The S2/S1 ratio was 1.02. As the sizes were not altered before and after the lyophilization, the re-dispersibility of the solid was confirmed.

### 2.4. The Effect of UA and UA–PM on SH-SY5Y Cell Viability

**Figure 6:**
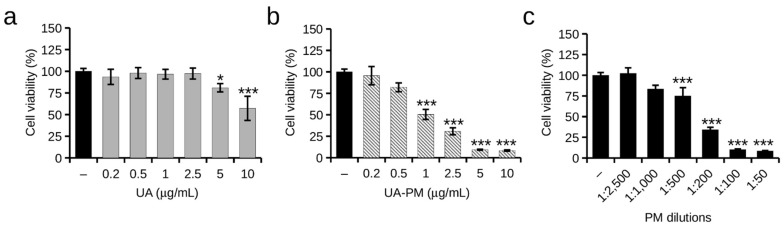
Effect of UA and UA–PM on the viability of SH-SY5Y cells. Cells were treated for 24 h with (**a**) UA at doses ranging from 0.2 to 10 µg/mL, (**b**) UA–PM at concentrations ranging from 0.2–10 µg/mL of loaded UA, and (**c**) corresponding dilutions of vehicle PM. Values are expressed as percentages compared with untreated control cells (−). Data are reported as mean ± SD of three independent experiments. Tukey’s test (*n* = 3). * *p* < 0.05, *** *p* < 0.001 vs. untreated control cells.

The effect of free UA and UA–PM on the SH-SY5Y cell viability cells was determined using an MTT assay. UA was assayed in a range of 0.2 to 10 µg/mL concentrations. As illustrated in Figure 6a, UA showed no signs of cell toxicity at the lower doses (0.2–2.5 µg/mL), while UA had cytotoxic effects at 5 µg/mL and caused about a 40% (57 ± 13%) reduction in viability at the concentration of 10 µg/mL compared with the untreated control cells. The cells treated with UA–PM showed a 50% (50 ± 5.9%) reduced viability already at the 1 µg/mL UA–PM concentration (Figure 6b). The cytotoxic effect of UA–PM observed in the SH-SY5Y cells was attributed to the toxicity of the vehicle, whose surfactant-rich composition can cause damage even at high dilutions of the cellular structures (Figure 6c). Indeed, this finding agrees with our previous work in which polymeric micelles of Soluplus–Solutol HS15 were used in an experimental model of SH-SY5Y [44]. Then, subsequent experiments were carried out using UA–PM at concentrations of loaded UA that were nontoxic to the cells, i.e., 0.2 and 0.5 µg/mL, and corresponding to the nontoxic dilutions of the vehicle itself.

### 2.5. The Effect of UA and UA–PM on SH-SY5Y Cell Migration

Among the various biological activities of UA, the literature attributes anticancer properties to the dibenzofuran derivative [55], including anti-migratory and anti-invasive abilities in some in vitro cancer cell models [18,19].

Some anticancer agents are cytotoxic with different sensitivities to actively growing cancer cells compared with non-cancer cells. However, anticancer agents are also generally toxic to non-cancer cells, thus causing adverse effects in cancer patients. In the process of metastasis, migrating and invasive cancer cells spread throughout the body, causing secondary sites of invasion and damage to organs and tissues [56]. Since migration is a hallmark of cancer cells, blocking or slowing the migration of cancer cells without cytotoxic effects and in a cell-safe manner represents a therapeutic strategy to counteract cancer more specifically and safely.

**Figure 7:**
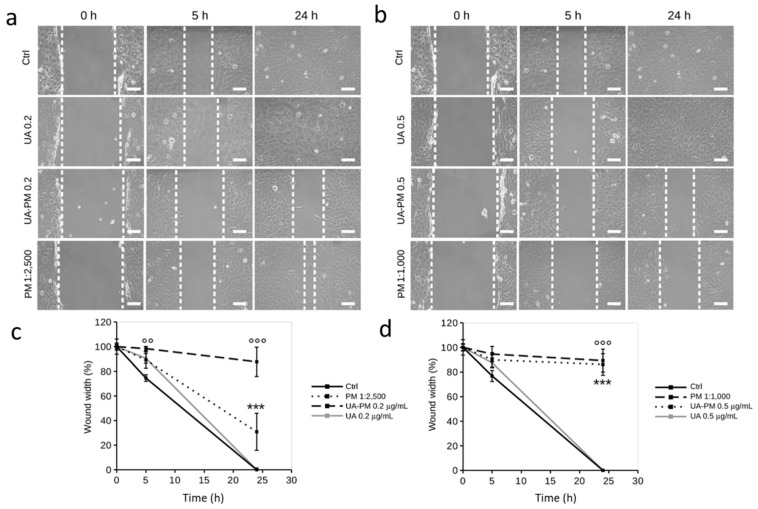
Effect of UA and UA–PM on the migration of SH-SY5Y cells. (**a**,**b**) Representative image of the migration of SH-SY5Y cells untreated (−) or after treatments with UA (0.2 and 0.5 μg/mL), UA–PM (0.2 and 0.5 μg/mL), and PM (1:2500 and 1:1000). Scratch closure was monitored for 24 h. The edges of the wound area are indicated by dashed lines. (**c**,**d**) Analysis of wound closure over time in SH-SY5Y cells treated under various experimental conditions. ImageJ image analysis software was used to measure and quantify wound width at each time point. Data are representative of three different experiments. Error bars represent standard deviation. *** *p* < 0.001 vs. untreated control cells; °° *p* < 0.01, °°° *p* < 0.001 vs. UA-treated cells.

In this work, the potential inhibitory effect of UA on the human neuroblastoma SH-SY5Y cell migration was evaluated by wound healing assay using nontoxic concentrations of free UA (0.2 and 0.5 µg/mL).UA had no significant effect on cell migration (Figure 7a,b); the cells treated with UA (0.2 µg/mL and 0.5 µg/mL) migrated across the wound width to complete closure at 24 h after the initial scratch with a migratory phenotype comparable to that of the untreated control cells (Figure 7c,d).

Since mixed polymeric micelles of Soluplus–Solutol and TPGS polymeric micelles are a largely used nanoformulation for biopharmaceutical studies [57,58], here, the ability of PM to promote an anti-migratory role of UA on SH-SY5Y cells was evaluated using doses of UA loaded in micelles loaded corresponding to those of free UA.

Concentrations of 0.2 and 0.5 µg/mL of UA–PM corresponded to noncytotoxic dilutions of vehicle PM (1:2500 and 1:1000, respectively) that ruled out the possible interference of PM toxicity on cell migration. As shown in Figure 7a,b, there was a slowing of migration of UA–PM-treated cells at both concentrations as early as 5h after the initial scratch. Notably, this effect was significant in cells treated with UA–PM 0.2 µg/mL that maintained a wound width of 98 ± 1.7% compared with untreated control cells (74 ± 2.7%). The inhibitory effect of UA–PM at both doses of UA was maintained over time; in fact, at 24 h after the initial scratch, wound width was 87 ± 11% and 89 ± 9% in cells treated with UA–PM 0.2 and 0.5 µg/mL, respectively (Figure 7c,d).

To rule out any potential effect of the vehicle on SH-SY5Y cell migration, PM at the two dilutions (corresponding to UA–PM 0.2 and 0.5 µg/mL) was tested. It is noteworthy that PM 1:2500 resulted in a slight reduction in cell migration while keeping a wound width of 30 ± 15% at 24 h after the initial scratch, whereas PM 1:1000 totally contributed to the inhibition of cell migration. In fact, at 24 h after the initial scratch, the cells treated with PM 1:1000 maintained a wound width of 86 ± 9% (Figure 7c,d).

When taken together, these data highlight that UA loading in PM allows UA to exert its anti-migratory role at doses that are nontoxic (0.2 µg/mL) to cells. Thus, we can speculate that PM increases UA activity by affecting not only UA solubility but also its cellular permeability.

### 2.6. The Effect of UA and UA-MP on MMP-2/9 Gelatinolytic Activity

ECM degradation is an important step in the cell migration process, allowing cells to migrate out of the primary tumor and metastasize. MMPs, particularly MMP-2/9 (or gelatinases), are involved in ECM degradation playing a key role in cell migration [59]. Targeting metalloproteases, and thus cell migration and metastasis formation, is thus a challenge for more effective anticancer therapy [60,61]. In this context, the effect of UA–PM on the gelatinolytic activity was examined by gelatin zymography assay. Based on the results of the wound healing assay, the activity of MMP-2/9 was evaluated in the culture media collected after 24 h of treatment with UA–PM 0.2 µg/mL, which was capable of significantly inhibiting cell migration. The role of free UA was tested to confirm the potential enhancement of the PM vehicle on UA bioactivity. The cells treated with the PM vehicle 1:2500 were used as a control.

**Figure 8:**
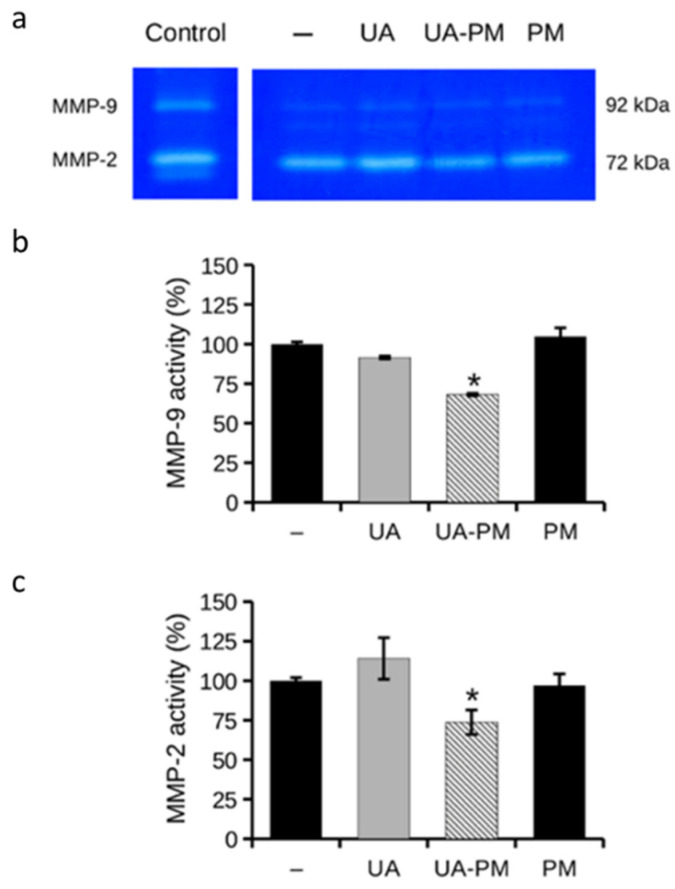
Effect of UA and UA–PM on MMP-2/9 release in culture medium. (**a**) Representative image of gelatin zymography of cell culture media collected at 24 h from SH-SY5Y cells untreated (−) or treated with UA (0.2 μg/mL), UA–PM (0.2 μg/mL), and PM (1:2500). Culture medium of untreated HT1080 human fibrosarcoma cells was used as a control for the molecular size of MMPs (Control). Quantitative data of gelatinolytic bands of (**b**) MMP-9 and (**c**) MMP-2. Data are reported as means ± SD from three different experiments. * *p* < 0.05 vs. untreated control cells. Tukey’s test (*n* = 3).

As shown in Figure 8a, the gelatin zymography assay showed that UA–PM was able to significantly reduce MMP-2 and MMP-9 activity by about 30% (73 ± 7.8% and 68 ± 0.7%, respectively) compared with the untreated control cells (Figure 8b,c). It is noteworthy that the neuroblastoma cell lines, including the SH-SY5Y cells, generally secrete negligible amounts of MMP-9 [62]; however, the inhibitory effect of UA–PM on MMP-9 secreted in the culture medium was found to be statistically significant.

These data suggest that UA–PM can act on cell migration and inhibit the gelatinolytic activity of the SH-SY5Y neuroblastoma cells. Other natural compounds with migrastatic action on human neuroblastoma cells have demonstrated improved efficacy when loaded into nanocarrier [44,63]. Thus, it is possible to suggest a potential application of UA delivered in polymeric micelles to counteract the metastatic migration of cancer cells.

## 3. Materials and Methods

### 3.1. Chemicals and Reagents

Badische Anilin-und Soda Fabrik (BASF, Ludwigshafen, Germany) kindly provided Soluplus and Solutol HS15. The Merck Millipore’s Simplicity^®^ UV Water Purification System was used to obtain distilled water (Merck KGaA, Darmstadt, Germany). Sigma-Aldrich (Milan, Italy) provided (+)-usnic acid (purity 98%), Phosphate-buffered saline pH 7.4 (PBS), D-α-Tocopherol polyethylene glycol 1000 succinate (TPGS), Methanol HPLC grade, Acetonitrile HPLC grade, Formic acid analytical grade, Dichloromethane (CH_2_Cl_2_) and dimethyl sulfoxide (DMSO). Phosphotungstic acid was purchased from Electron Microscopy Science (Hatfield, MA, USA). The cell culture material was purchased from Merck KGaA (Darmstadt, Germany), including Ham’s F-12 Nutrient Mixture, Dulbecco’s Modified Eagle Medium (DMEM), Fetal Bovine Serum (FBS), L-glutamine, antibiotics (penicillin and streptomycin), 3-(4,5-Dimethylthiazol-2-yl)-2,5-Diphenyltetrazolium Bromide (MTT), Coomassie Brilliant Blue G-250, gelatin, and all other chemicals and solvent for the in vitro tests. Bio-Rad supplied the electrophoresis reagents (Hercules, CA, USA). Sarstedt (Nümbrecht, Germany) supplied the disposable plastics.

### 3.2. Preparation of Polymeric Micelles (PM) and Usnic Acid-Loaded Polimeric Micelles (UA–PM)

The thin-film hydration method was employed to prepare empty PM [40,44,64]. Briefly, Soluplus, Solutol HS15, and TPGS (1:4:0.5 weight ratio) were dissolved in a 10 mL CH_3_OH/CH_2_Cl_2_ mixture (1:4 *v*/*v*). The solvents were evaporated under vacuum, and the film was hydrated with 5 mL of deionized water while being sonicated for 3 min to form a micellar dispersion. UA was added in the organic phase to prepare UA–PM, obtaining a final concentration of 0.5 mg/mL.

The optimization of the formulation involved numerous tests with different polymer ratios and different UA concentrations. Soluplus:TPGS in ratio 4:1, 3:2, 2:3, Soluplus:Solutol:TPGS 1:4:1 and 1:4:0.5 were tested. UA was loaded from 0.5 to 1 mg/mL. Based on the physical characteristics, loading capacity, and stability of the micelles, Soluplus, Solutol, and TPGS 1:4:0.5 weight ratio was selected. The amount of solvent was related to the previously published studies [40,42,44,47].

### 3.3. Characterization of PM and UA–PM

The average diameter, size distribution (polydispersity index, PDI), and zeta potential values were determined using a Zeta Sizer Pro instrument (Malvern Instruments Ltd.; Worcestershire, UK) at 25 °C. The results are reported as the mean of the three measurements.

The encapsulation efficiencies (EE%) were determined using the dialysis method. Two mL of the samples were inserted in a previously hydrated dialysis bag (cut-off 12–14 kDa). The bag was placed in a beaker containing 1 L of water and kept under constant magnetic stirring for one hour at room temperature. After that, the contents of the bag were withdrawn and diluted to 1:50 with methanol, and the sample was placed for 30 min in an ultrasonic bath and ultracentrifuged at 14,000× *g* rpm for 10 min. Finally, the supernatant was collected and analyzed by HPLC-DAD analysis.

The amount of encapsulated UA was determined using the HPLC-DAD method, with the mobile phase containing (A) formic acid/water with pH 3.2 and (B) acetonitrile. The flow rate was set to 0.5 mL/min. The column was a Kinetex C18 (150 × 4.6 mm, 5 μm), maintained at 25 °C. The gradient profile was: 0.10–2 min 75–85% B, 2–4 min 85–90% B, 4–6 min 90–95% B, 6–8 min 95–100% B. The acquisition of the chromatographic profiles was carried out at a wavelength of 280 nm. The t*_R_* for UA was 7.28 min. Different concentrations ranging from 0.002 µg/µL to 0.99 µg/µL were used for the calibration curve. The linear correlation coefficient was >0.999.

The UV-vis measurements were performed using a UV/Vis 1900 Shimadzu Spectrophotometer (Tokyo, Japan). The samples were DMSO solutions of free UA (0.5 μg/mL), UA–PM (UA concentration 0.5 μg/mL), and PM in water.

### 3.4. Morphological Characterization

The samples were analyzed using the Scanning Electron Microscope Gaia 3 (Tescan s.r.o, Brno, Czech Republic) FIB-SEM (Focused Ion Beam-Scanning Electron Microscope). The electron beam used for the TEM imaging had a voltage of 15 kV, and it was operated in a high-vacuum mode and with a Bright-field TEM detector. The samples (10 μL) were applied to a 150-mesh carbon film-covered copper grid. After that, 5 μL of phosphotungstic acid solution (1% *w*/*v* in water) was dropped onto the grid as a staining medium, and the excess solution was removed using a filter paper. The samples were dried for 3 min, after which they were examined with the electron microscope and photographed at an accelerating voltage of 20 kV.

### 3.5. Theoretical Critical Micellar Concentration (CMC_theor_)

The following equation was used to calculate the theoretical CMC (*CMC_theor_*) value for PM [37]: *X_Soluplus_*, *X_Solutol_*, and *X_TPGS_* are the molar fractions of Soluplus, Solutol, and TPGS, and *CMC_Soluplus_*, *CMC_Solutol_*, and *CMC_TPGS_* are the CMC values of Soluplus, Solutol, and TPGS, respectively. The molar fractions were determined from the ratio between the moles of the constituent and the total moles of the constituents of the mixture.

(1) $$\frac{1}{CMC_{Theor}}=\frac{X_{Soluplus}}{CMC_{Soluplus}}+\frac{X_{Solutol}}{CMC_{Solutol}}+\frac{X_{TPGS}}{CMC_{TPGS}}$$

### 3.6. Lyophilization

UA–PM were frozen with liquid nitrogen and placed in a freeze-drier Leybold Heraeus Lyovac GT2 (Leybold GmbH, Cologne, Germany) at −20 °C. The lyophilized sample (UA–PML) was kept at room temperature in a silica gel desiccator until the analysis. The powder was redispersed with the original amount of deionized water and shortly vortexed to ensure its complete dispersion. The average particle hydrodynamic diameter, homogeneity, surface charge, and EE% were evaluated.

### 3.7. Storage Stability Studies

The physical and chemical stability of UA–PM as a colloidal dispersion and as a freeze-dried product (UA–PML) was investigated. The micellar dispersion was placed in glass bottles with plastic caps and stored at 4 °C for 10 days, while the freeze-dried powder was kept at room temperature for 30 days. At set times, the size, PDI, and zeta potential were evaluated by diluting UA–PM with deionized water. The chemical stability was checked to monitor the EE%. At 10, 20, and 30 days, UA–PML was solubilized in deionized water, and the physical (size, PDI, and zeta potential) and chemical (EE%) parameters were checked. The appearance and re-dispersibility of the powder were evaluated.

### 3.8. In Vitro Release Study

An in vitro release test was conducted to evaluate the percentage of UA release from UA–PM compared to the UA acetonitrile solution. In both cases, 2 mL of the sample, containing 0.5 mg/mL of UA, was placed inside a previously hydrated dialysis bag (regenerated cellulose, Spectrum Laboratories Inc., Breda, The Netherlands, MWCO 12–14 kDa). Then, the bag was inserted in a beaker containing 200 mL of release medium consisting of PBS:EtOH 70:30 and placed under magnetic stirring at a temperature of 37 °C. To maintain the sink conditions, 1 mL of the release medium was withdrawn and replaced with an equal volume of the PBS:EtOH mixture at predetermined intervals. The collected aliquots were analyzed by HPLC-DAD to determine the amount of UA released during 24 h. All of the experiments were carried out in triplicate.

### 3.9. Cell Line and Culture Condition

The ATCC (American Type Culture Collection) provided the SH-SY5Y human neuroblastoma cells (ATCC^®^ No. CRL-2266TM). The cells were grown in a 50% Ham’s F12 and 50% DMEM supplemented with L-glutamine (2 mM), streptomycin (100 μg/mL), penicillin (100 U/mL), and 10% FBS (complete medium) in a humidified 5% CO_2_ incubator. At 90% confluence, the SH-SY5Y cells were trypsinizated (0.25% trypsin, 0.5 mM EDTA) and propagated. The following in vitro cell-based experiments were conducted in a heat-inactivated serum medium (HI-FBS medium). HI-FBS was obtained by incubating FBS at 55 °C for 30 min.

### 3.10. Cell Viability

The viability of the SH-SY5Y cells was analyzed using the MTT colorimetric assay. Briefly, the cells were seeded in 96-well plates (5 × 10^3^ cells/well) in a complete medium. The cells were then treated with UA (0.2–10 µg/mL) and UA–PM (0.2–10 µg/mL of loaded UA) in a HI-FBS medium. As controls, the untreated cells and the cells treated with empty PM were used. The cells were incubated with MTT solution (0.5 mg/mL) at 37 °C in the dark for 1 h. After washing with PBS, the insoluble formazan crystals were dissolved by adding 100 µL/well of dimethyl sulfoxide. The iMARK microplate reader was used to measure the absorbance values (Bio-Rad, Hercules, CA, USA) at a wavelength of 595 nm. The data obtained from a biological triplicate are reported as percentages compared with the untreated control cells.

### 3.11. Wound Healing Assay

SH-SY5Y cell migration was evaluated under various experimental conditions using a wound healing assay [65]. The cells were seeded in 6-well plates (5 × 10^5^ cells/well). A 200-µL sterile plastic tip was used to wound the monolayer longitudinally, and the following PBS washing removed the nonadherent cells. The SH-SY5Y cells were then treated with UA (0.2 and 0.5 μg/mL) and UA–PM (0.2 and 0.5 μg/mL of loaded UA) in a HI-FBS medium for 24 h. The untreated cells and the PM-treated cells were used as controls. Phase-contrast microscopes with a digital acquisition system (Nikon TS-100, Digital Sight DS Fi-1, Nikon, Minato-ku, Tokyo, Japan) were used to observe the cell-free area (at 0, 5, and 24 h after scratching). Wound width was measured and quantified by ImageJ image analysis software version 1.53e (National Institutes of Health, Bethesda, MD, USA).

### 3.12. Gelatin Zymography Assay

The gelatinolytic activity of MMP-2/9 was assessed using gelatin zymography, as previously described [65]. Briefly, the cells were seeded in 24-well plates (12 × 10^4^ cells/well) and incubated overnight. The cells were then treated with UA (0.2 μg/mL) and UA–PM (0.2 μg/mL of loaded UA) in HI-FBS medium for 24 h, while the untreated cells and the cells treated with empty PM were used as controls.

Cell debris was removed by centrifuging the culture supernatants at 9700× *g* at 4 °C for 1 min. The conditioned medium (2 μL/sample) was electrophoresed in an 8% polyacrylamide gel containing 1 mg/mL gelatin under nonreducing conditions. The 24-h-conditioned medium of the human fibrosarcoma HT1080 cells was used as a standard control activity of MMP-2/9 [66].

To remove SDS, the gel was washed twice (30 min each) in 2.5% (*v*/*v*) Triton X-100 before being incubated for 30 min at room temperature in reaction buffer (50 mM Tris-HCl pH 7.4, 0.2 M NaCl, 5 mM CaCl_2_, 1 mM ZnCl). Overnight incubation was performed in the reaction buffer. The gel was then incubated for 1 h at room temperature with a solution of 40% (*v*/*v*) methanol and 10% (*v*/*v*) acetic acid for protein fixation. Following two 10-min washes in distilled water, gel staining was performed with 0.05% colloidal Coomassie Brillant Blue G-250 dissolved in 1.6% (*v*/*v*) phosphoric acid, 8% (*w*/*v*) ammonium sulfate, and 20% (*v*/*v*) methanol. Gelatinase activity was visible as clear bands on a blue background after the staining was removed with 1% (*v*/*v*) acetic acid. The zymography images were captured using a digital scanner.

### 3.13. Statistical Analysis

One-way analysis of variance (ANOVA) followed by Tukey’s HSD test was used to analyze the in vitro data. The threshold for statistical significance was set at *p* < 0.05.

## 4. Conclusions

This study investigated the potential applicability of PM containing Soluplus, Solutol, and TPGS as a delivery system to increase the solubility of UA and its anti-migratory activity in human neuroblastoma SH-SY5Y cells. PM proved to be an excellent formulation by presenting good physical parameters, high encapsulation efficiency, and ensuring prolonged release. The freeze-drying process prolongs the formulation’s storage stability, preventing aggregation and/or chemical degradation.

In addition, in vitro cell-based experiments showed that nontoxic doses of UA–PM inhibited cell migration compared with the same doses of free UA. UA–PM also had an inhibitory effect on the gelatinolytic activity of MMP-2/9, known to be involved in the process of cell migration. Thus, polymeric micelles influence not only the solubility of UA but also its anti-migratory activity promoting, via the polymeric constituents, permeability across cell membranes. Overall, this study sheds light on polymeric micelles as an optimal formulation to deliver lipophilic drugs that can increase their solubility and permeability. Further in vivo studies will be necessary to prove the efficacy of UA delivered in polymeric micelles as a migrastatic agent.

## Figures and Tables

**Table 1:** Physical and chemical characterization of empty (PM) and UA-loaded polymeric micelles (UA–PM). Data are reported as mean ± SD of *n* = 3 experiments.

Sample	Size (nm)	PDI	Zeta Potential (mV)
**PM**	58 ± 0.23	0.19 ± 0.00	−6.44 ± 0.36
**UA–PM**	45 ± 0.17	0.26 ± 0.00	−14.24 ± 1.75

## Data Availability

Data is contained within the article and Supplementary Materials.

## Supplementary Materials

The following supporting information can be downloaded at: https://www.mdpi.com/article/10.3390/ph15101207/s1. Figure S1. UV-Vis spectra of UA solution in DMSO (1), UA–PM (2), and PM (3), the concentration of UA was 5 μg/mL.
