# Supplementary figures and images for: Usnic Acid-Loaded Polymeric Micelles: An Optimal Migrastatic-Acting Formulation in Human SH-SY5Y Neuroblastoma Cells
Source: Pharmaceuticals (Basel). 2022 Sep 29;15(10):1207. doi: 10.3390/ph15101207 (PMC9607998; doi:10.3390/ph15101207)

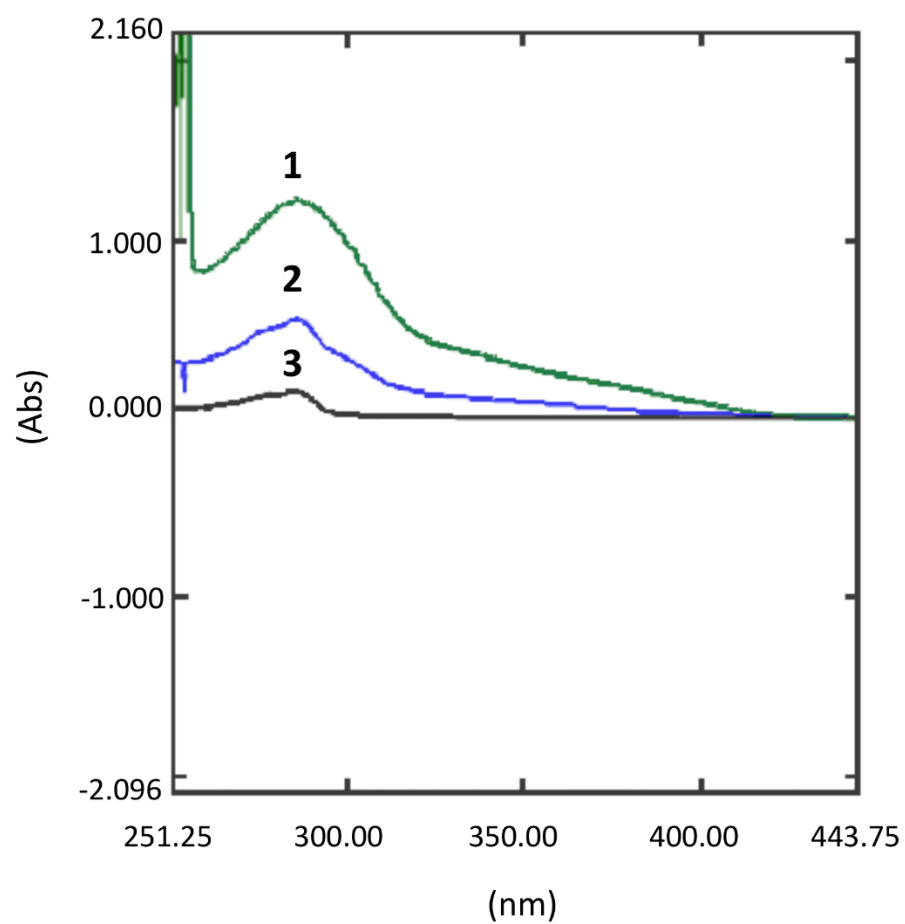

**Figure S1.** UV-Vis spectra of UA solution in DMSO (1), UA-PM (2), and PM (3), the concentration of UA was 5 mg/mL.

Supplement: Supplementary file 1 [file pharmaceuticals-15-01207-s001.zip › pharmaceuticals-1916839-supplementary.pdf]
